# Emerging Artificial Intelligence Tools for the Screening of Structural and Valvular Heart Disease

**DOI:** 10.1007/s11897-026-00757-w

**Published:** 2026-05-13

**Authors:** Yasmine Abbaoui, Alexis Nolin-Lapalme, Julianne Morisset, Ines El Adib, Philippe Genereux, Timothy J. Poterucha, Pierre Elias, Xioaxi Yao, Robert Avram

**Affiliations:** 1 Faculty of Medicine, University of Montreal, Montreal, QC Canada; 2 HeartWise.Ai, Montreal Heart Institute, Montreal, QC Canada; 3 Gagnon Cardiovascular Institute, Morristown Medical Center, Morristown, NJ USA; 4 Department of Cardiology, Mayo Clinic, Rochester, MN USA; 5 Department of Biomedical Informatics, Columbia University, New York, NY USA; 6 Seymour, Paul and Gloria Milstein Division of Cardiology, Columbia University Irving Medical Center and NewYork-Presbyterian Hospital, New York, NY USA; 7 Division of Health Care Policy and Research, Department of Health Sciences Research, Mayo Clinic, Rochester, MN 55905 USA; 8 Robert D. and Patricia E. Kern Center for the Science of Health Care Delivery, Mayo Clinic, Rochester, MN USA; 9 Department of Cardiovascular Medicine, Mayo Clinic, Rochester, MN USA; 10 Division of Cardiology, Department of Medicine, Montreal Heart Institute, 5000 Belanger, Montreal, QC H1T 1C8 Canada

**Keywords:** Artificial intelligence, Heart failure, Structural heart disease, Valvular heart disease, Low left ventricular ejection fraction, Screening

## Abstract

**Purpose of Review:**

Structural heart disease (SHD) encompasses diseases involving the heart valves, chambers, walls, and muscles. Current diagnostic methods have limited accessibility and predictive value. This review aims to present recent advances in artificial intelligence (AI)-guided tools in the screening of SHD and valvular heart disease (VHD), and to present challenges and opportunities for their use in clinical practice.

**Recent Findings:**

AI-guided models trained on ECGs, chest X-rays, and coronary artery calcium scans have a high accuracy in the diagnosis of SHD, heart failure, low left ventricular ejection fraction, and VHD. Some of these models can highlight the signals that influence their predictions, improving explainability.

**Summary:**

The use of AI in screening for SHD and VHD could lead to earlier diagnosis, enhanced accuracy, and better accessibility. However, outcome data on earlier diagnosis using these tools is required before broad deployment.

**Supplementary Information:**

The online version contains supplementary material available at 10.1007/s11897-026-00757-w.

## Introduction

Structural heart disease (SHD) is defined as non-coronary cardiac disease involving the valves, walls, chambers, or muscles. It includes valvular heart diseases (VHD), low left ventricular ejection fraction (LVEF), defined as LVEF ≤ 40% [1], left ventricular hypertrophy (LVH), left and right cardiac dysfunction and pulmonary hypertension (PHTN) [2]. It is responsible for a significant proportion of cardiovascular diseases, such as heart failure (HF) and will progress if left untreated.

More than 64 million people worldwide live with HF [3], with a global cost estimated at more than 284 billion US dollars in 2021. Despite the introduction of novel treatment for SHD, patients often remain untreated or treated too late to affect outcomes. Moreover, true prevalence of SHD is underestimated. In the PREVUE-VALVE study, the prevalence of moderate-to-severe VHD among individuals aged 65 to 85 who were systematically screened in pharmacies reached 8.2%. This number is almost twice as high as previously reported, suggesting that many patients remain unaware of their condition [4, 5]. Early screening is thus essential. Current screening methods are based on clinical history, auscultation, N-terminal pro-B-type natriuretic peptide (NT-pro-BNP), chest X-ray and echocardiography. However, these methods have limited predictive value. According to a systematic review, the sensitivity and specificity for the auscultation of heart murmurs varied between 16 and 91%, and 59 and 100%, respectively [6]. Similarly, NT-pro-BNP is not a specific biochemical marker [7, 8]. Trans-thoracic echocardiography (TTE), the gold standard method, requires an experienced sonographer to perform the test and a cardiologist to interpret the results, which can limit accessibility and lead to longer waiting times. A recent study found that only 48.1% of patients hospitalized for HF in Canada had access to an on-site echocardiography, and 28.7% did not have access to NT-pro-BNP testing [9]. This motivates the need for alternative diagnostic methods.

In the last few years, artificial intelligence (AI) has emerged with viable tools to guide SHD screening and its applications have increased exponentially [10], with the potential to detect asymptomatic diseases from low-cost tests such as electrocardiograms (ECGs). A recent study by Jabbour et al*.* showed that ECG-AI, a deep learning (DL) model trained on electrocardiograms, outperformed clinical risk scores in the detection of atrial fibrillation (AF) before the appearance of clinical signs of arrhythmia [11]. The use of AI-assisted screening methods could potentially help target high risk patients more efficiently and lower the hospitalization costs associated with SHD.

The aim of this non-systematic narrative review is to present recent studies related to AI-based screening of SHD, low LVEF, aortic stenosis (AS) and HF. It covers studies published in the last 6 years, to reflect the most recent trends in AI-related diagnostic tools and to discuss their limitations.

## Methods

### Literature Search Strategy

A comprehensive non-systematic literature search was conducted using Pubmed (MEDLINE), medRxiv, and arXiv in October 2025. The search strategy combined Medical Subject Headings (MeSH) terms and keywords related to: (1) AI methodologies, (2) cardiac pathologies, (3) screening or diagnostic, and (4) diagnostic tools. [Complete search strings and methodology provided in Online Resource 1].

### Eligibility Criteria

Studies were included if they: (1) evaluated AI-based tools (including machine learning (ML), DL, or any algorithmic model) for screening or diagnosis of SHD, reduced LVEF (< 50%), HF, or VHD; (2) utilized any input modality (echocardiography, electrocardiography, cardiac magnetic resonance imaging, computed tomography scan, clinical data, or multimodal inputs); (3) reported diagnostic/screening performance metrics; and (4) were published since 2019. Exclusion criteria included: conference abstracts without full-text availability, case reports, editorials, reviews without original data, studies focusing solely on treatment prediction or prognosis without diagnostic/screening endpoints, and duplicate publications.

### Study Selection

Two independent reviewers (YA, RA) screened titles and abstracts, followed by full-text review of potentially eligible studies. Out of the 84 studies screened, 30 were found eligible.

### Data Synthesis

Descriptive synthesis was performed given anticipated heterogeneity in AI architectures, input modalities, and outcome definitions.

## Results

**Fig. 1:**
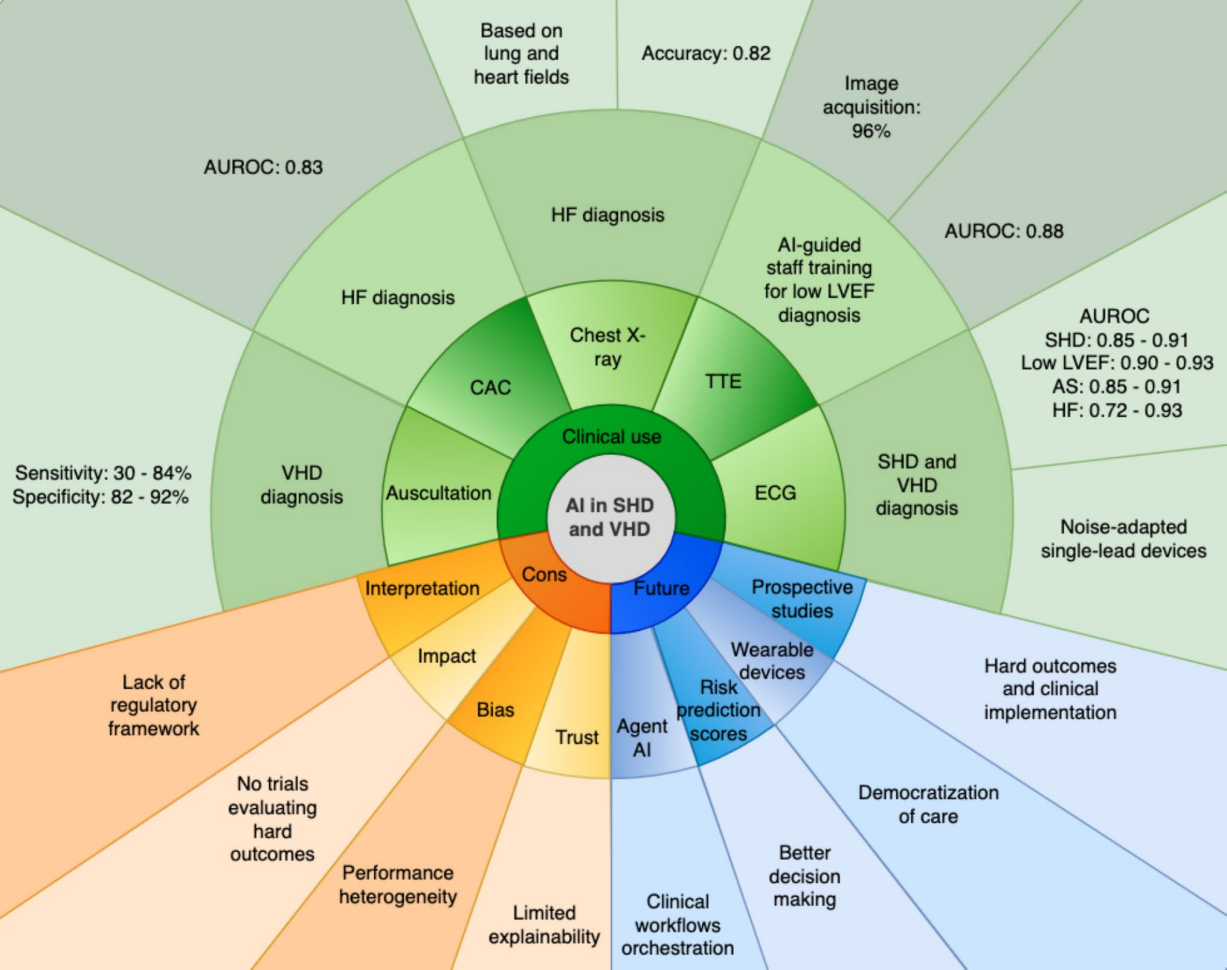
AI applications in SHD and VHD detection, a central illustration. AI: Artificial Intelligence; SHD: Structural Heart Disease; VHD: Valvular Heart Disease; CAC: Coronary Artery Calcium Scan; TTE: Transthoracic Echocardiography; ECG: Electrocardiogram; HF: Heart Failure; LVEF: Left Ventricular Ejection Fraction; AUROC: Area Under the Receiver Operating Characteristic Curve; AS: Aortic Stenosis

Results are presented by pathology and are summarized in Fig. 1 and Table 1.

**Table 1:** AI-based screening tools for structural heart disease: condensed table

First author et al	Algorithm name	Outcome	n	AUROC internal	AUROC external	Sens	Spec	AUPRC	PPV	NPV	Comments	FDA status
SECTION 1: STRUCTURAL HEART DISEASE (SHD)												
Ulloa-Cerna et al. [12]	rECHOmmend	SHD composite	> 2 M ECGs	0.91	N/S	90%	N/S	N/A	42%	N/S	41% had SHD within 1 yr; screening tool	Not FDA-cleared
Poterucha et al. [13]	EchoNext	SHD composite	> 1 M ECGs	0.852	0.80	72.6%	80.7%	0.785	N/S	N/S	AI > cardiologists; screening tool	Clinical trials ongoing (NCT06462989)
Dhingra et al. [14]	PRESENT-SHD	SHD composite	261 K ECGs	0.886	Consistent	89.8%	66.2%	0.807	57.4%	92.8	Works with smartphone photos; less accurate age ≥ 65; 2 - 4 × risk	Not FDA-cleared
Aminorroaya et al. [15]	ADAPT-HEART	SHD composite	267 K ECGs	0.879	0.859	90.9%	61.8%	N/A	54.6%	93.1%	Single-lead wearable; NNT 2 - 12; 3 - 6 × risk	Not FDA-cleared
SECTION 2: LOW LVEF												
Nolin-Lapalme et al. [16]	DeepECG-SSL	LVEF ≤ 40%	25 K ECGs	0.928	0.907	99.3%	45.4%	52.5%	54.5%	93.2%	Self-supervised; open-source; 10 external validations	Clinical trial ongoing (NCT06637293)
Khunte et al. [17]	Single-lead DL	LVEF < 40%	386 K ECGs	0.90	0.90	0.90	0.68	0.46	0.2	0.99	Noise-adapted for portable ECGs	Not FDA-cleared
Yao et al. [18]	AI-ECG	LVEF ≤ 50%	23 K pts	N/S	N/S	N/S	N/S	N/A	N/S	N/S	RCT; increased diagnosis OR 1.32	Not FDA-cleared
Attia et al. [19]	AI-ECG	LVEF ≤ 35%	3874 pts	0.918	N/S	82.5%	86.8%	N/A	N/S	N/S	Accuracy of 86.5%;	Not FDA-cleared
Lee et al. [20]	AiTiALVSD	LVEF ≤ 40%	96 pts	0.919	N/S	89.8%	94.0%	N/A	71.5%	98.2%	Better than NT-proBNP; TCAV analysis	Not FDA-cleared
Lee et al. [21]	ECG Buddy	LVEF < 40%	23 K ED	0.908	N/S	83.5%	83.0%	N/A	N/S	N/S	Image-based; multiethnic; beats NT-proBNP	Not FDA-cleared
Huang et al. [22]	AI-guided echo	LVEF < 50%	100 pts	0.880	N/S	84.6%	91.4%	N/A	78.5%	94.1%	Novice trained in 2 weeks	Not FDA-cleared
Attia et al. [23]	AI network	LVEF ≤ 35%	98 K pts	0.930	N/S	86.3%	85.7%	N/A	33.8%	98.7%	Accuracy of 85.7%; 4 × risk	Not FDA-cleared
Dhingra et al. [24]	AI-ECG	LVEF < 40%	231 K pts	0.718	N/S	89%	N/S	0.91	N/S	N/S	Beats PCP-HF; 4 - 24 × risk	Not FDA-cleared
Anumana (Rochester, Minnesota, USA) [25]	ECG-AI LEF	LVEF ≤ 40%	> 16 K ECGs	0.932	N/S	85.4%	83.6%	N/A	N/S	N/S	First FDA approval; reimbursement approved	FDA-cleared Oct 2023; DEPLOYED
Eko Health (emeryville, California, USA) [26]	ELEFT	LVEF < 40%	3456 ECGs	0.835	N/S	74.7%	77.5%	N/A	N/S	N/S	15-s detection with stethoscope	FDA-cleared Mar 2024; DEPLOYED
Tempus AI (Chicago, Illinois, USA) [27]	ECG-Low EF	LVEF ≤ 40%	N/S	N/S	N/S	N/S	N/S	N/A	N/S	N/S	Binary output; not for paced rhythms; positive dx in 2 in 5 pts with TTE	FDA-cleared Jul 2025; DEPLOYED
Bunkerhill (Carelog); (San Francisco, CA, USA) [28]	ECG-EF	Reduced LVEF	N/S	N/S	N/S	N/S	N/S	N/A	N/S	N/S	Carebricks platform integration	FDA-cleared Sep 2025; DEPLOYED
SECTION 3: VALVULAR HEART DISEASE												
Ghanayim et al. [29]	Vogx	AS	256 pts	N/S	N/S	84%	92%	N/A	N/S	N/S	AI-based stethoscope; less accurate in LFLG AS and inaudible murmurs	Approved
Roquemen-Echeverri [30]	Eko	VHD	1029 pts	N/S	N/S	39.3%	82.3%	N/A	N/S	N/S	AI-based stethoscope	Approved
Prince et al. [31]	Eko	Structural murmurs	615 pts	0.92	N/S	85.6%	84.4%	N/S	N/S	N/S	Outperforms clinicians	Approved
Cohen-Shelly et al. [32]	AI-ECG	Moderate-severe AS	259 K ECGs	0.85	N/S	78%	74%	N/A	N/S	99%	FP had 2.18 HR for AS in 15yrs	Not FDA-cleared
Popat et al. [33]	Meta-analysis	AS	20 studies	0.909	N/S	0.83	0.81	N/A	Low	N/S	ECG-based best; high heterogeneity	Meta-analysis
Elias et al. [34]	ValveNet	AS, AR, MR	77 K pts	0.880	0.76	78%	73%	0.33	20%	97.6%	NNS = 5; QRS most important	Not FDA-cleared
SECTION 4: HEART FAILURE PREDICTION												
Dhingra et al. [35]	AI-ECG	Incident HF	N/S	0.723	N/S	N/S	N/S	N/A	Similar to scores	N/S	Beats PCP-HF, PREVENT; 3 - 7 × risk	Not FDA-cleared
Matsumoto et al. [36]	CXR DL	HF	638 CXRs	N/S	N/S	N/S	N/S	N/A	N/S	N/S	Accuracy 0.82; no clinical history	Not FDA-cleared
Segar et al. [37]	oRSF	10—yr HF risk	N/S	0.880 (Black), 0.890 (White)	N/S	N/S	N/S	N/A	N/S	N/S	Race-specific superior	Not FDA-cleared
Naghavi et al. (preprint)	AI-CAC	15—yr HF	N/S	0.826	N/S	N/S	N/S	N/A	N/S	N/S	CAC-based; non-peer reviewed	Not FDA-cleared; Preprint

**Abbreviations: ***AUROC* area under the receiver operating characteristic curve, *AUPRC* area under the precision-recall curve, *Sens* sensitivity, *Spec* specificity, *PPV* predictive positive value, *NPV* negative predictive value, *FDA* US food and drug administration, *SHD* structural heart disease, *M* million, *ECG* electrocardiograms, *N/S* not stated, *N/A* not applicable, *AI* artificial intelligence, *K* thousand, *LVEF* left ventricular ejection fraction, *NNT* number needed to treat, *Pts* patients, *RCT* randomized controlled trial, *OR* odds ratio, *NT-proBNP* N-terminal pro-B-type natriuretic peptide, *TCAV* testing with concept activation vectors, *PCP-HF* pooled cohort equations to prevent heart failure, *TTE* echocardiography, *AS* aortic stenosis, *LFLG* low-flow, low-gradient, *VHD* valvular heart disease, *FP* false positive, *HR* hazard ratio, *AR* aortic regurgitation, *MR* mitral regurgitation, *NNS* number needed to screen, *HF* heart failure, *PREVENT* predicting risk of cardiovascular disease EVENTs, *CXR* chest X-ray, *DL* deep learning, *oRSF* optimized random survival forest, *CAC* coronary artery calcium

### AI in Structural Heart Disease

Several studies have validated AI-based models for predicting the risk of newly detected SHD on TTE. In 2022, Ulloa-Cerna et al. developed rECHOmmend, a DL model based on over 2 million ECGs, age, and sex to predict SHD risk (composite endpoint of moderate to severe VHD, LVEF ≤ 50% or interventricular septal thickness > 15 mm) at one year. The model had an area under the receiver operating characteristic curve (AUROC) of 0.91, with a positive predictive value (PPV) of 42% at 90% sensitivity. The use of a composite endpoint rather than individual disease-specific outcomes was shown to enhance prevalence, PPV, and overall diagnostic accuracy. Its accuracy decreased in patients with pre-existing HF and pacemakers. In their retrospective deployment, among 11% of patients without pre-existing SHD, 41% had SHD on echocardiography within 1 year [12]. Similarly, Poterucha et al*.* used more than one million ECGs to train EchoNext, a DL model pairing ECG, age and gender for risk prediction of SHD (composite endpoint of low LVEF ≤ 45%, maximum low left ventricular wall thickness ≥ 1.3 cm, moderate or severe right ventricular dysfunction, PHTN, moderate or severe VHD or a moderate or large pericardial effusion). The model had an AUROC of 0.85. It utilized 12-lead ECG as a cost-effective screening tool to identify individuals at high risk for SHD, thereby triaging patients who warrant confirmatory TTE—a more expensive and less accessible modality. In a reader study, 13 board-certified cardiologists evaluated 150 ECGs from the test set to assess human accuracy in detecting SHD using ECG alone, without clinical history or physical examination. Each cardiologist first reviewed ECGs showing only the waveform, standard ECG parameters (heart rate, intervals), patient age, and sex. Subsequently, the same ECGs were presented again with the addition of EchoNext's risk prediction score (0 - 1 scale) and categorical interpretation (score < 0.6: not consistent with SHD; ≥ 0.6: consistent with SHD) displayed on the interface. This "AI assistance" condition allowed cardiologists to integrate the algorithm's output into their decision-making. EchoNext achieved accuracy, sensitivity, and specificity of 77.3%, 72.6%, and 80.7%, respectively. Cardiologists without AI assistance demonstrated 64.0% accuracy, 61.1% sensitivity, and 66.1% specificity. When provided with AI assistance (i.e., seeing EchoNext's prediction alongside the ECG), cardiologists' performance improved to 69.2% accuracy, 64.7% sensitivity, and 72.4% specificity - though still inferior to the AI model alone. EchoNext demonstrated modestly superior discrimination in younger age groups [13]. These findings suggest that DL models, trained on paired ECG-echocardiography data, can extract latent electrocardiographic features imperceptible to human experts and may offload resource-intensive screening workflows by triaging patients for confirmatory imaging.

Moreover, beyond detection of SHD, ML can predict its progression. Dhingra et al*.* evaluated PRESENT-SHD, an ensemble extreme gradient boosting (XGBoost) model trained on 261,228 ECGs recorded 30 days before echocardiography to detect SHD defined as LVEF < 40%, moderate-or-severe left VHD or severe LVH. The AUROC for the test set was 0.886. Accuracy was maintained when using smartphone photographs of ECGs from monitors and printouts, but decreased in patients aged ≥ 65. A positive PRESENT-SHD screen was associated with a 2- to 4-fold increase in risk of new SHD or HF. The model demonstrated strong predictive discrimination in identifying the severe SHD phenotype. Every 10% increase in predicted risk led to a 36% higher likelihood of incident SHD or HF [14].

Additionally, Aminorroaya et al*.* introduced a single-lead ECG DL algorithm, ADAPT-HEART (Adapting Portable Technology in HEART disease detection), trained on 266,740 ECGs to predict SHD (composite of LVEF < 40%, moderate or severe left‑sided VHD and severe LVH) from wearable ECG signals. ADAPT-HEART had an AUROC of 0.879, and a high probability raised SHD risk 2.8–5.7-fold compared to low probability. Depending on the SHD prevalence, 2 to 12 patients would have to undergo echocardiography to detect one case of new onset SHD[15]. This approach enables deployment of AI-based screening and risk stratification for SHD using wearable ECG devices.

### AI in Reduced Left Ventricular Ejection Fraction

ECG-based AI for detecting left ventricular systolic dysfunction (LVSD) represents a widely adopted and regulated application, providing non-invasive, accessible screening for at-risk patients. Khunte et al*.* developed a noise-adapted single-lead DL model for the detection of low LVEF < 40%, with an AUROC of 0.90 for standard and noise-adapted models on standard ECGs. The noise-adapted model demonstrated improved performance on highly noisy portable ECGs, maintaining excellent discriminative ability even when recordings contained twice as much noise as signal [17].

DeepECG-SSL, a self-supervised learning model pre-trained using contrastive multi-segment coding, achieved an AUROC of 0.926 (95% CI: 0.923 - 0.929) with PPV of 54.5% and negative predictive value (NPV) of 93.2% for detecting LVEF ≤ 40%, and an AUROC of 0.887 (95% CI: 0.884 - 0.891) with PPV of 53.2% and NPV of 94.9% for detecting LVEF < 50% on the MHI-ds dataset. Interestingly, this self-supervised learning approach, similar to what was used to train generative pre-trained transformers such as ChatGPT, performed best on downstream tasks with limited annotated data such as pairing ECGs with TTE [16].

A recent study explored the transparency of the AiTiALVSD model for the detection of low LVEF ≤ 40% based on ECGs obtained 24 h prior to echocardiography. The model had an AUROC of 0.919, and its specificity decreased with ECG noise. Higher AiTiALVSD scores were associated with lower LVEF values. In a subset of 96 patients, AiTiALVSD had a better accuracy than NT-proBNP (AUROC of 0.905 versus 0.72, respectively), and sensitivity (92.6% versus 88.9%, respectively). The most important variables for risk prediction were left bundle branch block (LBBB), right bundle branch block, AF and conduction disorders [20]. Lee H. et al*.* validated ECG Buddy, an image-based AI model using 22,599 emergency department visits of patients from multiple ethnic groups in the US to detect LVEF < 40% on ECG. The AUROC, sensitivity, and specificity for the LVEF-ECG feature were 0.908, 83.5%, and 83.0%, compared with 0.740, 74.8%, and 62.0% for NT-proBNP, respectively [21].

In 2021, a randomized controlled trial (RCT) by Yao et al. enrolled 22,641 patients without HF to AI-ECG, a DL model applied to routine ECGs, versus usual care to compare identification of new low LVEF ≤ 50% within 90 days of ECG. In the overall patient population, there was an increase in diagnosis of low EF (2.1% in AI-ECG cohort versus 1.6% in the control cohort, odds ratio (OR) 1.32 (1.01–1.61), P = 0.007) and the same occurred in the positive AI-ECG population (14.5% in the control arm versus 19.5% in the intervention arm, OR 1.43 (1.08–1.91), P = 0.01). Although more TTEs were done for patients with positive AI-ECGs, there was no difference between the two groups in the number of TTEs obtained [18]. Despite strong diagnostic performance (AUROC 0.93 for LVEF ≤ 35%, PPV 19.5% in high-risk patients, NPV > 98%) [23], pragmatic RCTs of AI-ECG screening for low LVEF have demonstrated increased early detection rates (OR 1.32 - 1.50) and more targeted TTE utilization, but have failed to show improvements in hard clinical outcomes [18, 38].

AI can also be applied on TTEs. Huang et al*.* conducted a prospective cohort study in which a novice operator with no experience underwent two weeks of training to use AI-guided echocardiography to screen 100 patients for HF (LVEF < 50%). The novice produced interpretable images for 96 patients in an average time of 12 min 51 s, with an AUROC of 0.880. When compared with expert ultrasonographers’ techniques and cardiologists’ interpretations, the degree of agreement was 0.895, with a Cohen’s kappa of 0.742 ± 0.101 [22].

### AI in Valvular Heart Disease

Auscultation skills have declined, particularly for detecting regurgitation and moderate VHD [39, 40]. Digital acoustic data enables DL-based analysis, with AI-enhanced stethoscopes now commercially available: Sanolla VoqX for AS (sensitivity 84%, specificity 92%) [29], EKO AI murmur analysis software for VHD (sensitivity 39%, specificity 82%) [30], and validation studies showing pathologic murmur detection (sensitivity 86%, specificity 84%) [31].

The PREVUE-VALVE study (NCT05357404) revealed that AS affects 3.1% of Americans aged 65 - 85 years old with moderate or greater disease, representing approximately 1.8 million individuals, with prevalence escalating sharply with age (5.1% at 65 - 69 years to 14.7% at 80 - 85 years). Critically, many patients remain undiagnosed until symptomatic presentation or late-stage disease, when prognosis worsens substantially [4, 5]. Moreover, EARLY-TAVR demonstrated that early intervention in asymptomatic AS was superior to clinical surveillance and led to a reduction in the composite of death, stroke, or unplanned cardiovascular hospitalizations [41]. AI-ECG screening, through analysis of LVH, strain patterns, and voltage criteria, could facilitate timely surveillance TTE, risk stratification, and potentially earlier intervention in appropriate candidates, thereby preventing progression to irreversible myocardial damage and improving outcomes in a disease where therapeutic window is critical.

In 2021, Cohen-Shelly et al*.* trained an AI-ECG model on 258,607 ECGs performed within 180 days from a TTE to detect moderate to severe AS. The model had an AUROC of 0.85 with sensitivity, specificity, and accuracy of 78%, 74%, and 74%, respectively. Sensitivity increased and specificity decreased in older patients and men. Performance was better in patients with no comorbidities. When compared with true negatives, false-positive AI-ECGs had twice the risk of developing moderate to severe AS in 15 years (hazard ratio (HR) 2.18, 95% confidence interval 1.90 - 2.50). The NPV was 99%. The TP segment and the U wave in the right precordial leads had the biggest impact on the detection of AS. There was a positive correlation between probability of AS and its severity on echocardiogram [32].

In a meta-analysis of 20 studies, Popat et al*.* reported an AUROC of 0.909 for AI-based AS screening using ECG, auscultation, and imaging. Accuracy was higher with AI models based on ECGs than with other modalities (chest X-ray, electronic stethoscope, auscultation audio files, TTE, CT radiomics features of aortic valve calcium), with AUROCs of 0.924 versus 0.859, respectively. With a prevalence of moderate to severe AS of 4%, the PPV was low, which caused a possible risk for ordering unnecessary echocardiography. Performance was better for severe AS. However, the studies had a high degree of heterogeneity, caused by continent, AS type, data source, and AI-based approach type [33].

The previously described studies by Ulloa-Cerna et al. and Poterucha et al*.* found AUROCs of 0.908 and 0.864, for their rECHOmmend and EchoNext model, respectively, for the detection of moderate to severe AS [12, 13].

Elias et al*.* developed ValveNet, an ECG-based AI model with an AUROC of 0.880 for the detection of AS. The model performed well in both sexes, and race and ethnicity, with decreased accuracy in older patients and patients with a large QRS or a LBBB. The most important feature for the prediction of AS was the QRS complex, followed by the P waves, and variance in the R-R interval. In their screening program modeling analysis including patients aged 65 and older, five echocardiograms had to be performed in ValveNet ECG-positive patients to diagnose one moderate or severe left VHD (based on a set sensitivity of 75%, PPV of 21.5% and AS, aortic regurgitation, or mitral regurgitation prevalence level of 10%) [34].

### AI in Heart Failure

Recent studies have compared the predictive performance of traditional HF screening methods with that of AI models for the prediction of HF, with a particular emphasis on model explainability.

A retrospective study by Dhingra et al. validated an AI model predicting incident HF, defined as first HF hospitalization (HFH), based on single-lead ECGs, and compared its performance with Pooled Cohort Equations to Prevent Heart Failure (PCP-HF) and Predicting Risk of Cardiovascular Disease Events (PREVENT) risk scores. A positive AI-ECG result was associated with a 3- to 7-fold higher risk for HF. Every 10% increment in HF risk resulted in a 27 to 65% increase in hazard across cohorts, independently of age, sex, comorbidities, and competing risk of death. The AUROCs for AI-ECG, PCP-HF, and PREVENT were 0.723, 0.634 and 0.668, respectively. PPV was similar for the AI model and both risk scores. The addition of AI-ECG predictions to both risk scores improved C-statistic by 0.112 - 0.114 for PCP-HF and 0.080 - 0.101 for PREVENT [24].

Patients who receive a false positive result from AI-enabled ECG screening for low LVEF—defined as a positive AI-ECG screen but normal LVEF on TTE—have a significantly increased risk of subsequently developing new-onset HF compared to those with a negative screen, with HRs ranging from 3.9 to 24 across large multinational cohorts and up to a 4-fold increased risk in US health system data**.** This elevated risk persisted after adjustment for age, sex, comorbidities, and competing risk of death, indicating that a false positive AI-ECG may serve as an early digital biomarker for future HF, even in the absence of current LVSD [23, 24, 35]. The absolute incidence of new-onset HF in these false positive cases is not directly reported in all studies, but the association is consistent and robust across diverse populations and settings [42].

When examining other modalities, Matsumoto et al. trained a DL model on 638 chest X-rays to detect HF, defined as cardiomegaly or congestion. The model had an accuracy of 0.820, with heatmaps indicating that its decisions were based on the lung and heart regions. However, no clinical history was available for patients included in this study, and the model could have labeled cardiomegaly from hemodialysis or non-cardiac congestion as positive findings, which actually represent false positives [36].

In 2021, Segar et al. developed a race-specific ML model to predict risk of HF at 10 years, which had an AUROC of 0.880 in Black patients, and 0.890 in White patients. Accuracy was higher in the race-specific model than in models including race only as a covariate. In both races, NT-pro-BNP was an important risk factor for the prediction of HF. In Black patients, troponin, glycemic parameters, and socioeconomic factors were the most important variables, whereas in White adults, it was Cornell voltage, LVH, prevalent cardiovascular (CV) disease and traditional CV risk factors. The model with the highest accuracy was optimized Random Survival Forest (oRSF), which incorporated the top 20 most important predictors of HF risk [37].

A non-peer-reviewed preprint by Naghavi et al. studied AI-powered coronary artery calcium scans (AI-CAC) for the prediction of HF at 15 years based on cardiac chambers volumetry. The AI-CAC model outperformed NT-pro-BNP and the Agatston score, with AUROCs of 0.826, 0.741, and 0.712, respectively. Their model coloured the sections where enlarged cardiac chambers were detected [43].

These findings highlight AI’s capacity to extract prognostic signals across ECG, imaging, and clinical data, serving as early digital biomarkers for pre-clinical ventricular dysfunction.

### Clinical Implementation and Ongoing Outcome Trials

To address critical evidence gaps in AI-ECG screening outcomes, physician acceptability and real-world feasibility, three complementary trials are currently underway or planned. HEART-AI (NCT06462989) is an RCT evaluating whether AI-based risk stratification using a modified version of the EchoNext (trained using DeepECG-SSL) algorithm increases timely SHD detection compared with standard care, hypothesizing a doubling of diagnosis rates at 90 days. The trial compares AI-enhanced ECG triage against conventional clinician interpretation, with primary outcomes including detection yield and time to echocardiography.

**Fig. 2:**
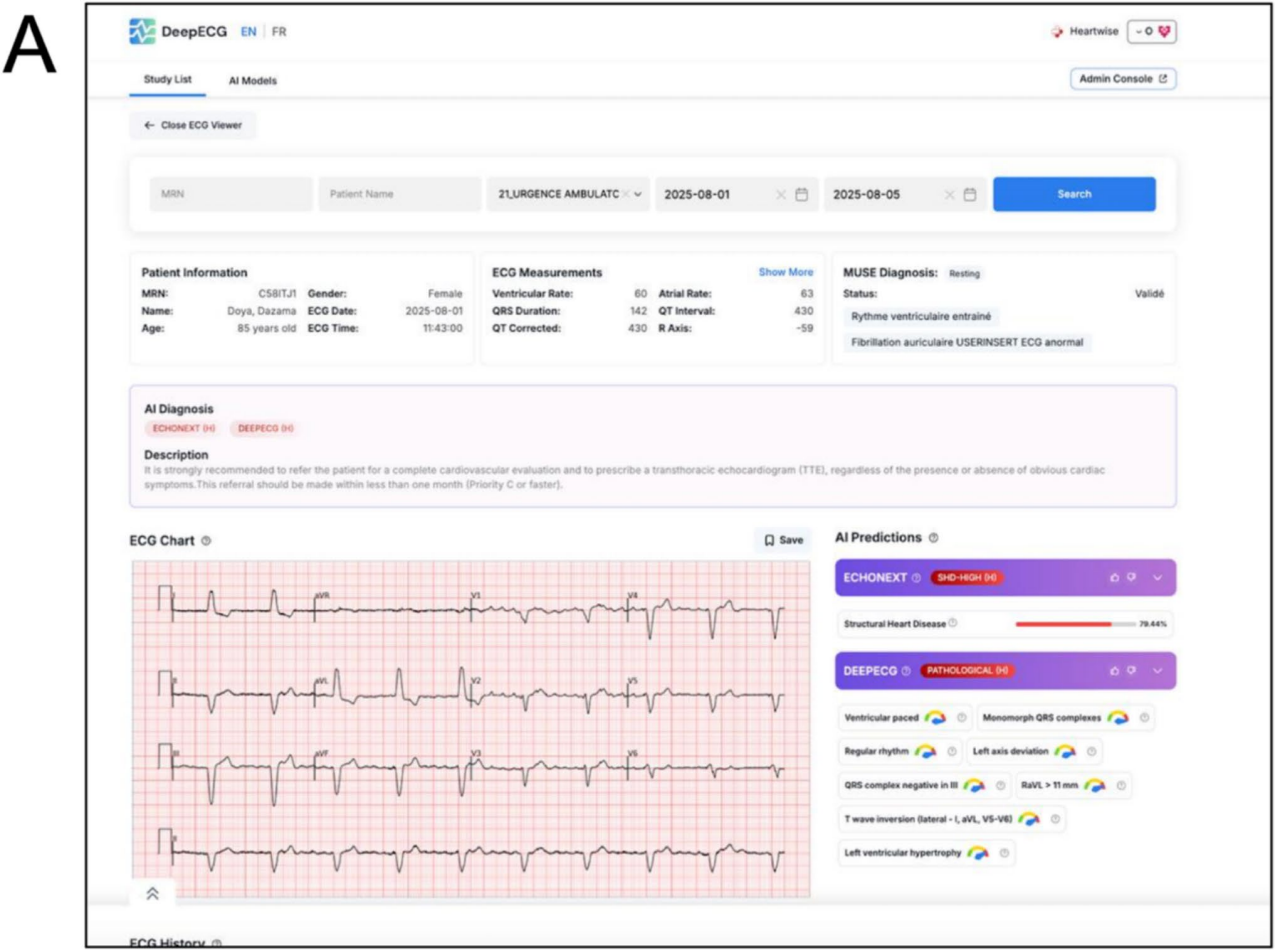
Screenshots of DeepECG.ai platform in the context of the DAISEA-ECG study. Legend A. Patient at high risk of structural heart disease and with an abnormal ECG, AI suggests rapid referral

DAISEA-ECG (NCT06637293) represents a pragmatic implementation of AI-ECG screening in primary care for cardiovascular risk stratification. The trial started recruitment in September 2025 and utilizes the DeepECG.ai platform, where XML files of 12-lead ECGs are automatically sent to our cloud-based platform to provide real-time interpretation (Fig. 2). The platform is accessible via an electronic health record (EHR) button and provides ECG interpretation and EchoNext risk prediction. It also recommends a referral if the ECG is abnormal (i.e., moderate to high risk of SHD or rhythm or ischemic abnormalities).

Critically, both trials incorporate linkage to provincial administrative databases for major adverse cardiovascular events (MACE) and incorporate patient and clinician surveys to assess AI clinical utility and acceptance barriers. The overarching objective is operationalizing AI into clinical workflows, improving diagnostic equity, and reducing avoidable echocardiography delays - which increase 1-year MACE risk 2.6-fold when exceeding 90 days (8.1% vs 3.1%).

The CACTUS trial (Project Number: 1R01HL177055 - 01) will look at patients ≥ 40 years old presenting to 8 emergency departments and undergoing ECGs. Those patients with high-risk AI-ECG scores, no prior history of SHD or TTE, and no plan for future TTE at the end of their clinical encounter will be randomized to usual care or AI intervention. Patients in the intervention arm will undergo outpatient echocardiography. The primary endpoint will be new SHD diagnoses at 90 and 365 days, and key secondary endpoints will be any class 1 or 2a changes in clinical management resulting from new SHD diagnoses.

These trials represent the critical next step in demonstrating whether AI-ECG screening translates diagnostic accuracy into improved hard patient outcomes.

### Future Directions: From Standalone Models to Agentic ECG-AI Systems

Current ECG-AI implementations function as isolated "widgets"—performing single diagnostic tasks (e.g., LVEF detection, rhythm classification) without integration into longitudinal care pathways or clinical decision support workflows. This narrow-scope deployment fails to capitalize on ECG-AI's potential to orchestrate downstream clinical actions. Agentic AI systems are large language model-driven frameworks that perceive, reason, and act iteratively upon their environment through tool use and memory. They offer a paradigm shift by embedding ECG-AI within adaptive clinical workflows [44]. Such systems could autonomously trigger context-appropriate order sets (e.g., initiating guideline directed medical therapy (GDMT), flagging patients for cardiology consultation), monitor longitudinal ECG trajectories to detect subclinical disease progression, and coordinate multidisciplinary care by interfacing with EHRs, scheduling systems, and communication platforms [45]. For instance, an agent detecting reduced LVEF could retrieve prior ECGs, assess progression rate, verify insurance authorization, schedule appropriate imaging, and notify the ordering physician, all while maintaining audit trails and human oversight checkpoints. However, regulatory frameworks for such autonomous, adaptive systems remain undefined, as existing medical device approval paradigms assume static, task-specific algorithms rather than dynamic, goal-directed ones.

### Controversies and Limitations

One of the challenges with the application of AI is the “black box” concept. In the EchoNext study, AI alone outperformed the combination of AI and clinicians, highlighting potential reluctance among clinicians to fully rely on AI predictions [13]. Patients could also struggle to understand the rationale behind their treatment - especially if AI recommends against it - if clinicians are unable to explain the reasoning behind AI-generated predictions. In this context, AI will most likely be used as a rule-in tool for heart disease screening, rather than a rule-out approach, as the consequences associated with underdiagnosis are more difficult to accept than unnecessary testing. Moreover, the use of AI does not appear to increase the total volume of screening tests, but instead helps identify high-risk individuals more precisely, thereby optimizing resource utilization [15, 18, 34]. Many studies are now exploring the variables that impact AI’s predictions, such as specific demographic variables or segments on the ECG known to play a role in the disease pathophysiology and commonly used by clinicians for diagnosis [20, 37]. This could improve the understanding and acceptance of AI as a guiding tool for clinicians. Preliminary cost-effectiveness modeling suggests potential value ($27,858-$43,351/quality-adjusted life year [QALY]) [46], especially in outpatient settings ($1,651/QALY). However, the absence of outcome data, such as mortality, HFH, or GDMT initiation, represents a critical evidence gap that must be addressed through adequately powered trials with extended follow-up before widespread clinical implementation can be recommended. Otherwise, early detection may merely shift diagnosis timing without altering disease trajectory [46]. The American Heart Association, in its 2024 scientific statement, recognizes that AI-ECG can detect ventricular dysfunction earlier than traditional testing and improve first detection rates, but also notes that prospective studies demonstrating improved patient outcomes are still needed [47].

Another issue is the lack of guidelines regarding the interpretation of AI predictions. The use of continuous values for risk predictions is difficult to interpret for clinicians, as it is an emerging tool, with few studies to back the use of such data for clinical decisions. As a result, dichotomous predictions accompanied by prescriptive directives, like, “high risk, order echocardiography” versus “low risk, no echocardiography needed unless strong clinical suspicion” are easier to use in a clinical setting. However, some AI models have demonstrated that higher risk scores are associated with an increased probability of disease incidence or severity [14, 15, 20, 24, 32]. Such models could serve as continuous risk scores to better characterize morbidity and mortality, providing additional clinical insights for physicians. In contrast, dichotomous predictions may lead to the loss of this valuable information.

Additionally, recent advances in wearables single-lead ECG technology could greatly enhance accessibility to cardiology care, enabling clinicians to remotely screen and monitor patients for heart disease, even in underserved or remote communities, provided portable devices are made financially accessible as well. However, most studies trained their models on one-lead data extracted from 12-lead ECGs rather than recordings from wearable, single-lead devices. Future studies should aim to validate these models on accessible wearable technologies to better reflect real-world application. It is important to note that AI should not replace clinicians’ judgment, but rather serve as an additional tool to support decision-making. Otherwise, ethical issues could arise regarding accountability and the burden of responsibility. The purpose of AI should not be to absolve clinicians of their decision-making duties, but rather to assist them in making better-informed choices using the most advanced tools available.

## Conclusion

AI-enabled ECG analysis demonstrates robust diagnostic performance for VHD and SHD (AUROC 0.88 - 0.93 for reduced LVEF), optimizing resource allocation through 2- to 12-fold enrichment in diagnostic yield without increasing total imaging volume. Self-supervised learning and multimodal integration further enhance accuracy and data efficiency. However, pragmatic RCTs show increased early detection rates, but no improvements in mortality, HFH, or GDMT initiation. This critical evidence gap - early detection without demonstrated clinical benefit - combined with limited model explainability and clinician trust barriers, precludes recommendation for widespread implementation. Adequately powered outcome trials with extended follow-up are essential before clinical deployment despite compelling diagnostic performance.

## Key References

- Poterucha TJ, Jing L, Ricart RP, Adjei-Mosi M, Finer J, Hartzel D, et al. Detecting structural heart disease from electrocardiograms using AI. Nature. 2025;644(8075):221-30. 10.1038/s41586-025-09227-0.
  - ○ A paper demonstrating that the EchoNext model outperforms cardiologists in the diagnosis of structural heart disease.
- Cohen-Shelly M, Attia ZI, Friedman PA, Ito S, Essayagh BA, Ko WY, et al. Electrocardiogram screening for aortic valve stenosis using artificial intelligence. Eur Heart J. 2021;42(30):2885-96. 10.1093/eurheartj/ehab153.
  - ○ This study shows that AI-ECG can serve as an effective tool for the prediction of moderate to severe aortic stenosis.
- Elias P, Poterucha TJ, Rajaram V, Moller LM, Rodriguez V, Bhave S, et al. Deep Learning Electrocardiographic Analysis for Detection of Left-Sided Valvular Heart Disease. J Am Coll Cardiol. 2022;80(6):613-26. 10.1016/j.jacc.2022.05.029.
  - ○ The manuscript illustrates that their ECG deep learning algorithm could be a powerful diagnosis tool for the detection of aortic stenosis, aortic regurgitation and mitral regurgitation.

## Supplementary Information

Below is the link to the electronic supplementary material.

Supplementary file1 (PDF 90 KB)

## Data Availability

No datasets were generated or analysed during the current study.
