# Supplementary material for: Emerging Artificial Intelligence Tools for the Screening of Structural and Valvular Heart Disease
Source: Curr Heart Fail Rep. 2026 May 13;23(1):23. doi: 10.1007/s11897-026-00757-w (PMC13171939; doi:10.1007/s11897-026-00757-w)
Supplement: Supplementary file 1 — Supplementary file1 (PDF 90 KB) [file 11897_2026_757_MOESM1_ESM.pdf]

Emerging Artificial Intelligence Tools for the Screening of Structural and Valvular Heart Disease

Current Heart Failure Reports

Yasmine Abbaoui[1,2], Alexis Nolin-Lapalme[1,2], Julianne Morisset[1,2], Ines El-Adib[1,2], Philippe Genereux[3], Timothy J. Poterucha[4], Pierre Elias[5,6], Xioaxi Yao[7], Robert Avram[1,2,8]

1) Faculty of Medicine, University of Montreal, Montreal, Quebec, Canada.
2) HeartWise.ai, Montreal Heart Institute, Montreal, Quebec, Canada.
3) Gagnon Cardiovascular Institute, Morristown Medical Center, Morristown, NJ, USA
4) Department of Cardiology, Mayo Clinic, Rochester, MN, USA.
5) Department of Biomedical Informatics, Columbia University, New York, NY.
6) Seymour, Paul and Gloria Milstein Division of Cardiology, Columbia University Irving Medical Center and NewYork-Presbyterian Hospital, New York, NY, USA.
7) Division of Health Care Policy and Research, Department of Health Sciences Research, Mayo Clinic, 55905 Rochester, MN; Robert D. and Patricia E. Kern Center for the Science of Health Care Delivery, Mayo Clinic, Rochester, MN; Department of Cardiovascular Medicine, Mayo Clinic, Rochester, MN.
8) Division of Cardiology, Department of Medicine, Montreal Heart Institute, Montreal, Quebec, Canada.

Correspondence: Robert Avram, 5000 Belanger, H1T 1C8, Quebec, Canada. (514)-376-3300. E-mail : Robert.avram.md@gmail.com

Detailed Search String and Data Extraction

The search strategy combined Medical Subject Headings (MeSH) terms and keywords related to (1) AI methodologies ("artificial intelligence" OR "machine learning" OR "deep learning"), (2) cardiac pathologies ("structural heart disease" OR "valvular heart disease" OR "heart failure" OR "left ventricular dysfunction" OR "reduced ejection fraction" OR "cardiomyopathies" or "heart diseases"), (3) screening or diagnostic applications ("screening" OR "diagnosis" OR "detection" OR "risk prediction"), and (4) diagnostic tools ("Physical Examination"[MeSH] OR "Electrocardiography"[MeSH] OR "Echocardiography"[MeSH] OR "Natriuretic Peptides"[MeSH] OR BNP[tiab] OR NT-proBNP[tiab] OR "B-type natriuretic peptide"[tiab] OR "clinical judgment"[tiab] OR "physical examination"[tiab] OR "history taking"[tiab] OR "bedside examination"[tiab] OR "clinical assessment"[tiab]). Only studies written in English, conducted on humans, and published since 2019 were screened. No restrictions on publication status were applied. Data extracted included: study characteristics (author, year, country, design, sample size), patient population demographics, AI model architecture, input data modality, target pathology (structural heart disease subtype, left ventricular ejection fraction threshold, valvular heart disease type and severity), reference standard, and performance metrics (area under the receiver operating characteristic curve, sensitivity, specificity, positive predictive value, negative predictive value, F1-score, accuracy).
